# 1-year survival rate of SARS-CoV-2 infected patients with acute respiratory distress syndrome based on ventilator types: a multi-center study

**DOI:** 10.1038/s41598-023-39992-9

**Published:** 2023-08-04

**Authors:** Ata Mahmoodpor, Kievan Gohari-Moghadam, Farshid Rahimi-Bashar, Masoum Khosh-Fetrat, Amir Vahedian-Azimi

**Affiliations:** 1 Research Center for Integrative Medicine in Aging, Aging Research Institute, Tabriz University of Medical Sciences, Tabriz, Iran; 2 Medical ICU and Pulmonary Unit, Shariati Hospital, Tehran University of Medical Sciences, Tehran, Iran; 3 Department of Anesthesiology and Critical Care, School of Medicine, Hamadan University of Medical Sciences, Hamadan, Iran; 4 Department of Anesthesiology and Critical Care, Khatamolanbia Hospital, Zahedan University of Medical Sciences, Zahedan, Iran; 5 Trauma Research Center, Nursing Faculty, Baqiyatallah University of Medical Sciences, Sheykh Bahayi Street, Vanak Square, P.O. Box 19575-174, Tehran, Iran

**Keywords:** Outcomes research, Paediatric research, Diseases, Medical research

## Abstract

The aim of this study was to evaluate the association between types of ventilator and the one-year survival rate of patients with acute respiratory distress syndrome (ARDS) due to SARS‑CoV-2 infection. This multi-center, retrospective observational study was conducted on 1078 adult patients admitted to five university-affiliated hospitals in Iran who underwent mechanical ventilator (MV) due to ARDS. Of the 1078 patients, 781 (72.4%) were managed with ICU ventilators and 297 (27.6%) with transport ventilators. Overall mortality was significantly higher in patients supported with transport ventilator compared to patients supported with ICU ventilator (16.5% vs. 9.3% *P* = 0.001). Regression analysis revealed that the expected hazard overall increased with age (HR: 1.525, 95% CI 1.112–1.938, *P* = 0.001), opacity score (HR: 1.448, 95% CI 1.122–2.074, *P* = 0.001) and transport ventilator versus ICU ventilator (HR: 1.511, 95% CI 1.143–2.187, *P* = 0.029). The Kaplan–Meier curves of survival analysis showed that patients supported with ICU ventilator had a significantly higher 1-year survival rate (*P* = 0.001). In MV patients with ARDS due to COVID-19, management with non-ICU sophisticated ventilators was associated with a higher mortality rate compared to standard ICU ventilators. However, more studies are needed to determine the exact effect of ventilator types on the outcome of critically ill patients.

## Introduction

The epidemic of Coronavirus disease-2019 (COVID-19), caused by Severe Acute Respiratory Syndrome-Coronavirus-2 (SARS-CoV-2), which was first discovered in Wuhan, China, quickly became a global pandemic^1,2^. Acute respiratory distress syndrome (ARDS), a life-threatening form of respiratory failure, is common among COVID-19 patients^3,4^. Evidence suggests that approximately one-third (33%) of hospitalized patients with COVID-19 develop ARDS, and approximately three-quarters (75%) of COVID-19 patients with ARDS are admitted to the intensive care units (ICUs), with a high proportion of them requiring mechanical ventilation (MV)^5,6^. The strategy of breathing support is crucial in treating ARDS due to COVID-19^7^.

The rapid increase in the numbers of patients with COVID-19 during pandemic, who require ICU care to receive MV has place healthcare systems around the world under enormous challenge and pressure^8,9^. The shortage of critical care resources, both in terms of equipment and trained personnel, has required the reorganization of hospital facilities, even in developed countries. Therefore, to accommodate the huge influx of patients, transient ICU beds have been created in operating rooms, emergency departments, and other parts of the hospital^10^. As a result, a significant proportion of patients with severe ARDS are being treated in hospital settings outside the ICU, using transport ventilators^11,12^.

Transport ventilators have been used for decades in emergency or transport scenarios, both within and outside of hospital^13,14^. However, the general view regarding these ventilators is that due to certain limitations, they are typically suitable for short-term usage, such as transportation, and their safety for providing prolonged ventilation to critically ill patients is questionable^13,15^. Undoubtedly, they have more limited capacities in terms of ventilation and monitoring modes compared to advanced ICU ventilators. However, disregarding their use in pandemic situation could restrict the expansion of treatment and care beyond the confines of the ICU walls for patients requiring MV^16^.

A single-center prospective observational study by Ferre et al.^17^, evaluated the association between ventilator type and hospital mortality in patients with ARDS related to COVID-19. They demonstrated that management in a transient ICU equipped with non-ICU sophisticated turbine-based ventilators was not associated with worse outcomes compared to a standard ICU equipped with ICU ventilators. However, they mentioned that the study design was not powered to demonstrate any differences in outcomes after adjustment. Furthermore, there is a lack of additional data examining clinical outcomes based on ventilator type and their impact on patients with ARDS due to COVID-19. In addition, the COVID-19 pandemic remains active worldwide, and more data from evidence-based studies involving diverse populations would be highly beneficial. Therefore, we conducted this multi-center observational study among hospitalized Iranian patients with COVID-19-related ARDS to compare the effects of ICU ventilator and transport ventilator on one-year post-CIVID-19 survival.

## Results

### Patients’ characteristics

A total of 1,078 patients with ARDS due to COVID-19, who underwent MV, were enrolled in this study. The mean age of the study population was 52.96 ± 14.46 years, and 773 (68.4%) patients were men. Only 36 (3.3%) of the patients had underlying diseases. Out of these 36 patients, 28, 7 and 2 subjects had cardiac, respiratory and kidney failure, respectively. The mean ± SD score of APACHE II and MV duration (hours) were 15.40 ± 2.28 and 250 ± 109.1, respectively. According to registry records, 29 (2.7%) patients had a history of re-admission after discharge. The lesion type of 414 patients (38.4%) was only GGO, 133 (12.3%) was GGO plus crazy paving, 42 (3.9%) had consolidation only, and 489 (45.4%) had GGO plus consolidation. In 383 patient, other findings such as linear opacity (n = 174, 45.4%), reversed halo sign (n = 49, 12.8%), pleural effusion (n = 55, 14.4%), intera lisional bronchiectasis (n = 61, 15.9%), and lymphadenopathy (n = 44, 11.5%) were observed. The mean opacity score of the study population was 6.16 ± 5.55.

### Comparison according to types of ventilator

Demographic, clinical and outcome data for all participants according to the types of ventilator are presented in Table 1. Of the 1078 patients, 781 (72.4%) were managed with ICU specialized ventilators, and 297 (27.6%) were managed with transport ventilators. The two groups were not significantly different in terms of age (*P* = 0.836), gender (*P* = 0.058), severity of illness based on APACHE II score (*P* = 0.321), history of readmission (*P* = 0.097), lesions distribution (*P* = 0.487), lesions type (> 0.05), underlying diseases (*P* = 0.189), presence diffuse opacity (*P* = 0.124), and total opacity score (*P* = 0.089). However, intera lesional traction bronchiectasis (8.4% vs. 4.6%, P = 0.016) and the number of involved lobes were significantly higher in the patients managed with transport ventilators compared to the patients supported by ICU specialized ventilator. MV duration was significantly lower in the patients supported with transport ventilators compared to patients in the ICU ventilator group (238.02 ± 106.55 vs. 254.53 ± 109.7, *P* = 0.026). Overall mortality (16.5% vs. 9.35, *P* = 0.001), in-hospital mortality (1.7% vs. 0.9%, *P* = 0.004) and out-of-hospital mortality (14.8% vs. 8.5%, *P* = 0.004) were significantly higher in the patients supported with transport ventilator compared to patients supported with ICU ventilator.

**Table 1:** Demographic, clinical and outcome characteristics according to ventilator types.

Variables	Total patients (n = 1078)	ICU ventilator (n = 781)	Transport ventilator (n = 297)	*P*-value
Age				
Mean ± SD	52.96 ± 14.46	53.02 ± 14.2	52.82 ± 15.06	0.836
Gender (%)				
Male (%)	737 (68.4)	521 (66.7)	216 (72.7)	0.058
Female (%)	341 (31.6)	260 (33.3)	81 (27.3)	
APACHE II				
Mean ± SD	15.40 ± 2.28	15.44 ± 2.25	15.28 ± 2.33	0.321
Re-admission (%)				
Yes	29 (2.7)	21 (2.7)	8 (2.7)	0.997
Lesions distribution (%)				
Bilateral + Multifocal	931 (86.4)	678 (86.8)	253 (85.2)	0.487
Others	147 (13.6)	103 (13.2)	44 (14.8)	
Lesions type (%)				
GGO	414 (38.4)	298 (38.2)	116 (39.1)	0.786
GGO + crazy paving	133 (12.3)	104 (13.3)	29 (9.8)	0.113
Consolidation	42 (3.9)	29 (3.7)	13 (4.4)	0.615
GGO + Consolidation	489 (45.4)	350 (44.8)	139 (46.8)	0.558
Other findings (%)				
None	695 (64.5)	511 (65.4)	184 (62)	0.897
Liner opacity	174 (16.1)	124 (15.9)	50 (16.8)	0.703
Reversed Halo sign	49 (4.5)	39 (5)	10 (3.4)	0.252
Pleural effusion	55 (5.1)	44 (5.6)	11 (3.7)	0.198
Interalesional traction bronchiectasis	61 (5.7)	36 (4.6)	25 (8.4)	0.016*
Lymphadenopathy	44 (4.1)	27 (3.5)	17 (5.7)	0.093
Underlying diseases (%)				
None	1041 (96.6)	760 (97.3)	281 (94.6)	0.189
Pulmonary	7 (0.6)	4 (0.5)	3 (1)	
Cardiac	28 (2.6)	16 (2)	12 (4)	
Kidney	2 (0.2)	1 (1)	1 (0.3)	
Presence diffuse opacity (%)				
Yes	181 (16.8)	122 (15.6)	58 (19.5)	0.124
No	897 (83.2)	659 (84.4)	239 (80.5)	
Number of involved lobe (%)				
No lobe involved	897 (83.2)	658 (84.3)	239 (80.5)	0.142
1	6 (0.6)	4 (0.5)	2 (0.7)	0.550
2	49 (4.5)	39 (5)	10 (3.4)	0.001*
3	50 (4.6)	31 (4)	19 (6.4)	0.001*
4	43 (4)	30 (3.8)	13 (4.4)	0.001*
5	33 (3.1)	19 (2.4)	14 (4.7)	0.001*
Total opacity score				
Mean ± SD	6.14 ± 5.55	5.95 ± 5.31	6.60 ± 6.13	0.089
MV duration				
Mean ± SD	250 ± 109.1	254.53 ± 109.7	238.02 ± 106.55	0.026*
Outcomes (%)				
Live	956 (88.7)	708 (90.7)	248 (83.5)	0.001*
Death	122 (11.3)	73 (9.3)	49 (16.5)	
In-hospital mortality	12/122 (9.8)	7 (0.9)	5 (1.7)	0.004*
Out-of-hospital	110/122 (91.2)	66 (8.5)	44 (14.8)	

GGO: Ground-glass opacities, APACHE: Acute Physiology and Chronic Health Evaluation (APACHE) II, * *P* < 0.05 was statistically significant.

### Proportional hazard Cox regression findings

**Figure 1:**
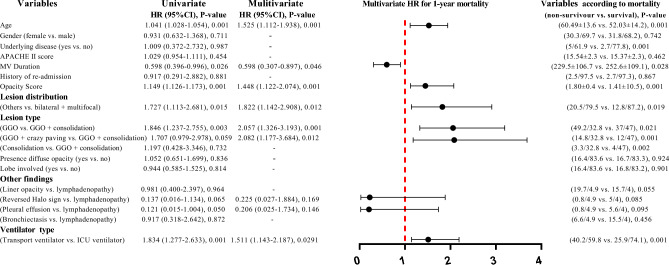
Univariate and multivariate Cox regression model to predict the 1-year mortality according to demographic, clinical, chest CT characteristics and ventilator type. To avoid over-fitting in the multivariate model, just the variables which leads to *p*-value ≤ 0.2 in univariate analysis, were entered to multivariate model. Forest plot showed hazard ratio for 1-year mortality of multivariate analysis. In addition, according to outcome (mortality vs. live status) we defined two groups as non-survival and survival, which variables were compared according these groups.

Univariate and multivariate Cox regression model were used to predict 1-year mortality, using demographic, clinical, chest CT characteristics, and ventilator type (Fig. 1). Multivariate Cox regression analysis revealed that the expected hazard 1-year mortality increased with higher age (HR: 1.525, 95% CI 1.112–1.938, *P* = 0.001), a higher opacity score (HR: 1.448, 95%CI: 1.122–2.074, *P* = 0.001), other lesion distribution versus bilateral + multifocal distribution (HR: 1.822, 95% CI 1.142–2.908, *P* = 0.012), GGO (HR: 2.057, 95% CI 1.326–3.193, *P* = 0.001), GGO + crazy paving (HR: 2.082, 95%CI: 1.177–3.684, *P* = 0.012), consolidation (HR: 1.631, 95% CI 0.564–4.711, *P* = 0.367), types of lesion versus GGO + consolidation, and transport ventilator versus ICU ventilator (HR: 1.511, 95% CI 1.143–2.187, *P* = 0.029). However, a higher MV duration can decrease the risk of mortality (HR: 0.598, 95% CI 0.307–0.897, *P* = 0.046).

### Survival analysis

**Figure 2:**
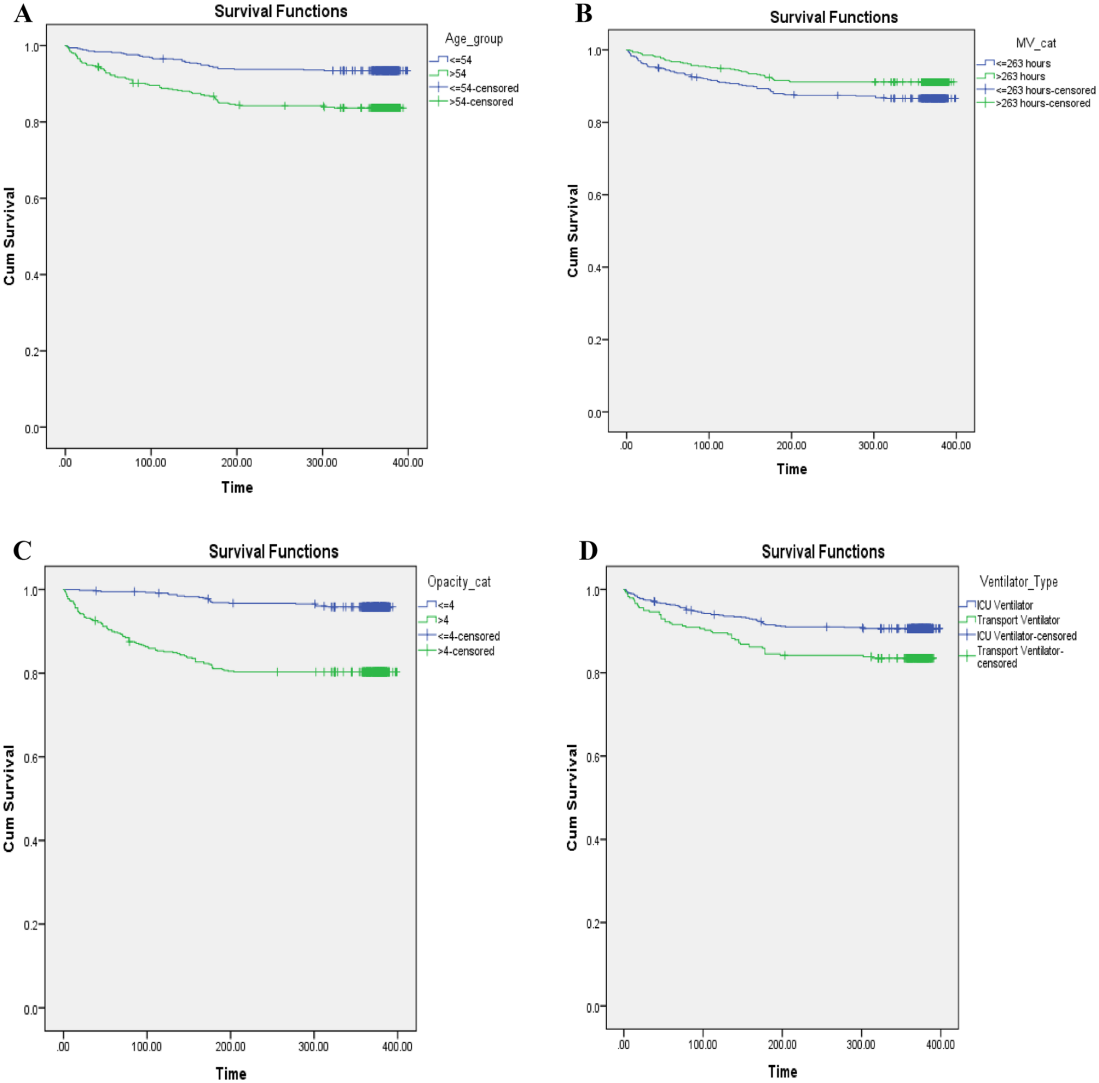
Kaplan–Meier curves estimated 1-year survival according to (**A**) age group (≤ 54 vs. > 54 years), (**B**) MV duration (≤ 263 vs. > 263 h), (**C**) total opacity score (≤ 4 vs. > 4) and (**D**) type of ventilator (ICU ventilator vs. transport ventilator).

The median follow-up time was 374 (363–380) days (mean: 339.4 ± 95.33 days). By the end of the study, 122 (11.3%) patients had died, and 956 patients (88.7%) were censored. The Kaplan–Meier (KM) curves of one-year survival according to age group (≤ 54 vs. > 54 years), MV duration (≤ 263 vs. > 263 h), total opacity score (≤ 4 vs. > 4) and type of ventilator (ICU ventilator vs. transport ventilator) are shown in Fig. 2A–D, and the survival time was compared among groups using the Log-Rank test. According to the results, patients over the age of 54 years (*P* < 0.001), those with a long-term MV duration (P = 0.018), patients with a lower (≤ 4) opacity score (*P* < 0.001) and patients supported with an ICU ventilator (P = 0.001) had significantly higher 1-year survival.

## Discussion

To our knowledge, this is the largest study that compares the effect of ventilator type on the outcome of critically ill patients with ARDS due to SARS COV-2 during the pandemics. In mechanically ventilated patients with ARDS due to COVID-19, management with non-ICU sophisticated ventilators was associated with a higher mortality rate compared to standard ICU ventilators. The surge in patients with COVID-19 requiring hospitalization, ICU admission, and ventilator support has posed an unparalleled challenge to physicians, nurses, hospital managers, and healthcare systems. Due to the limitation and shortage of critical care equipment in ICUs, a considerable proportion of patients who need MV are treated with transport ventilators in hospital settings outside the ICU^10,17^. However, despite the widespread use of transport ventilators in medical centers, their impact on COVID-19 patients' outcomes is unclear. To address this lack of information, a multi-center, observational study was conducted to evaluate one-year post-COVID-19 survival according to ventilator types among Iranian patients admitted for COVID-19-related ARDS requiring MV during the first wave of the COVID-19 pandemic.

In the current study, the mortality rate was significantly higher in patients supported with transport ventilators compared to patients supported with ICU ventilators. According to multivariate Cox regression analysis, age, opacity score, and the use of transport ventilators were associated with a higher risk of mortality. Our findings suggest that COVID-19 patients with a higher opacity score and GGO or GGO plus crazy paving lesion type had a lower survival rate. GGO is a sign of the early and active stage of the disease that can progress and worsen patients' health, while the appearance of consolidation opacities is a sign of proper activity of the immune system. Moreover, CT scan images showing GGO and GGO with consolidation, combined with COVID-19 pathological findings including hyaline membrane formation and increased inflammatory exudate in the alveolar space, are associated with increased mortality in patients^18–20^. However, a longer duration of mechanical ventilation (MV) was associated with a lower risk of mortality. Our hypothesis is that patient deaths may lead to a shorter use of mechanical ventilation, whereas a longer MV duration with professional ventilator settings would be safer for the patients. Additionally, this longer duration of MV may have resulted in extubation after the end of virus shedding and/or clinically significant lung inflammation, which is safer^21^. Furthermore, Kaplan–Meier curves from the survival analysis showed that patients supported with ICU ventilators had a significantly higher one-year post-COVID-19 survival rate.

The mortality rate in our study of patients requiring MV is lower than that reported in previous studies^17,22,23^. The majority of our patients (72.4%) were admitted to the ICUs, and the differences in mortality rates may be attributed to variations in strict criteria for ICU admission, such as oxygen requirements equal to or greater than 6–8 L/min to achieve a peripheral oxygen saturation of ≥ 90–92%, respiratory failure, shock, acute organ dysfunction, and patients at high risk for clinical deterioration. Moreover, the availability of ICU beds played a role, as non-emergency activities and surgeries in selected hospitals were rapidly reduced from the onset of the outbreak, and the number of ICUs beds for COVID-19 patients was increased^24,25^. Additionally, our lower mortality could be partially explained by the comparatively younger average age of our patients^26^.

Our results showed a significantly lower one-year survival among patients supported by transport ventilators. Transport mechanical ventilator models cannot reflect all the complexities of patient-ventilator interactions over time. Therefore, they do not provide an accurate estimate of pulmonary indices for physicians^16^. Moreover, monitoring lung indices during MV and adjusting ventilator settings based on them is crucial for achieving positive outcomes. Since transport ventilators have fewer monitoring capabilities compared to standard ones, this could be another reason for the higher mortality rate associated with these ventilators^27^. One of the most significant and independent indicators of mortality is mechanical power, which can be inaccurately estimated and lead to inappropriate ventilator support. One the other hand, patients in these scenarios typically have a high respiratory drive and require a higher level of flow, which may not be adequately provided by transport turbine-based ventilators^28,29^. This can result in increased agitation and desynchrony, leading to worse outcomes. However, in critical situation such as COVID-19 outbreak, when no ICU ventilators are available, the use of sophisticated turbine-based transport ventilators to treat patients who need MV is inevitable.

During the first wave of the COVID-19 outbreak, when no specific effective treatment against COVID-19 had been introduced, the majority of treatments relied on assisted ventilation, other organ-supportive interventions, and symptomatic anti-inflammatory medications^30,31^. Furthermore, at that time, there was limited knowledge about the optimal ventilation methods for these patients, and there was no time for training and learning. Therefore, in such critical situations, having professional operators who are familiar with all types of ventilators becomes crucial for the successful management of ventilator support and the successful weaning of patients from mechanical ventilation, ultimately leading to longer patient survival^32^.

**Figure 3:**
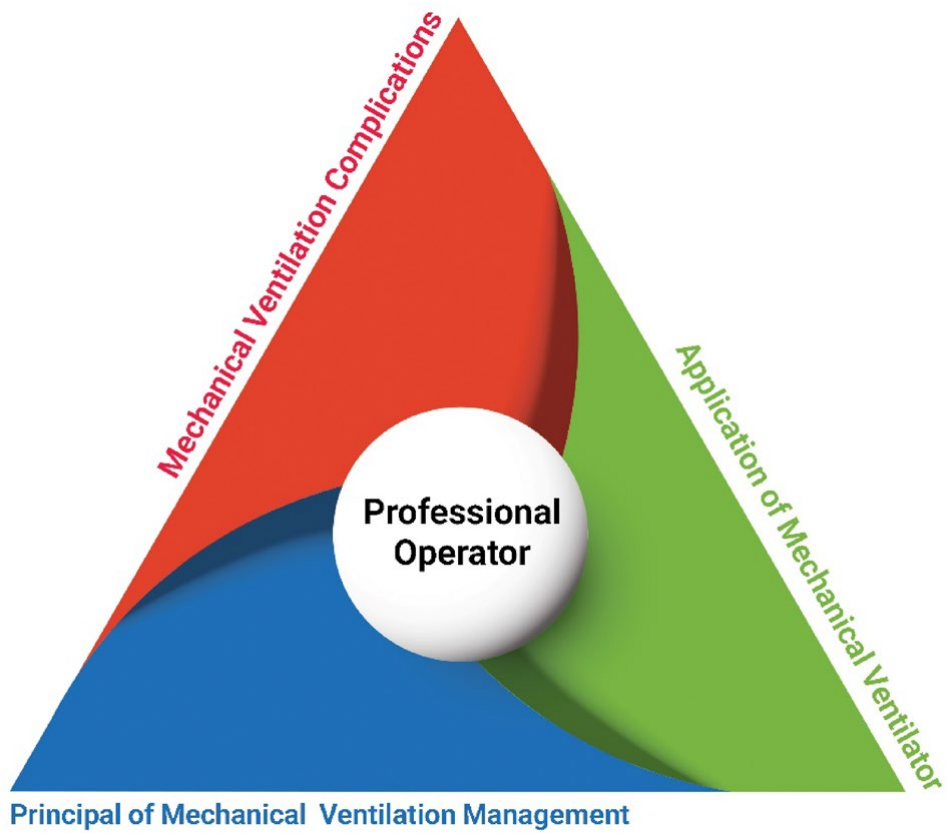
Triangle of professional operator as a key determinant in mechanical ventilation management.

The large influx of patients requiring mechanical ventilation for ARDS during the COVID-19 pandemic has necessitated the utilization of ventilators from a variety of sources, and healthcare workers should be familiar with these devices and their limitations. Optimization of standard critical care support is the best strategy to improve these patients' survival. The optimum results for the management of ventilator support and weaning from mechanical ventilation have three basic pillars, in which the professional operator is a key determinant (Fig. 3). Certainly, the critical care team in ICUs consisting of intensivists, critical care nurses, and respiratory therapists is more efficient and experienced in using equipment support devices (e.g., ventilators) and managing these patients than other healthcare workers outside of ICUs. Different ventilators have different capabilities, which could be unfamiliar to healthcare workers. Optimizing the target ventilation can depend on the ventilator type and the operator's decision regarding the management of the respiratory system situation. A study by Ferre et al.^17^, on ICU-admitted patients with COVID-19-related ARDS showed that the choice of ventilator type (ICU ventilator versus transport ventilator) not only depends on the situation of patients and the type of ventilators but also on the lack of experienced and expert staff who can address the limitations of these transport ventilators. Results of a similar study by Raymonds et al.^33^, showed that the mortality risk of ARDS patients was considerably higher in non-university compared with university hospitals. This study emphasized the importance of the trained and skilled physician and professional operator as important factors in mechanical ventilation management.

In order to enhance patient survival, during the shortage of ICU ventilators, clinicians should focus on increasing the optimal use of portable ventilators. This can be achieved through various strategies. Firstly, it is crucial to provide comprehensive training to healthcare professionals regarding different types of ventilators and their usage^34–36^. This will ensure that clinicians have the necessary knowledge and skills to effectively support patients in critical care settings. Additionally, given the limited understanding of ventilating COVID-19 patients, it is essential to offer guidance on the optimal settings, modes, and strategies for ventilator use^37,38^. Emphasizing a low tidal volume strategy and practicing lung-protective ventilation are particularly important for patients with acute respiratory distress syndrome (ARDS), as these approaches have been proven to enhance outcomes while reducing complications associated with ventilator use^39,40^. It is also vital to adhere to evidence-based guidelines and protocols to ensure appropriate ventilation support and minimize harm. Regular monitoring and evaluation of patients' response to ventilation, including factors such as oxygenation levels, lung compliance, and other relevant parameters, should be conducted^41,42^. Moreover, interdisciplinary collaboration among healthcare teams involving respiratory therapists, critical care specialists, and other knowledgeable professionals is crucial, especially considering the shortage of experienced staff and experts in transport ventilators^41,43^. By implementing these measures, clinicians can increase the optimal use of ventilators, ultimately leading to improved patient survival rates.

## Strengths and limitations

To our knowledge, this is the largest study comparing the effect of different types of ventilators on the outcomes of critically ill patients with ARDS caused by SARS-CoV-2 during a pandemic. The strengths of this study include its multicenter design and large sample size. However, the study also has some limitations. First, data collection was done during the peak of the COVID-19 pandemic, making it challenging to collect certain clinical data for these patients, as well as various laboratory parameters at different study centers, it was not possible to collect some clinical data for these patients. Second, it was not feasible to examine all the associated comorbidities related to COVID-19 in this study, and only three cases of cardiac, respiratory, and renal failure that were uniformly assessed in all participating patients were reported. Third, one of the predictive factors for survival in COVID-19 patients is age, and in this study, the age range of patients was middle-aged, which may limit the generalizability of these findings compared to Western countries where patients tend to be older. However, to prevent confounding factors, both univariate and multivariate analyses were used, and the severity of illness was assessed using the APACHE (Acute Physiology and Chronic Health Evaluation) II score, which provides an assessment of illness severity by assigning scores based on various physiological parameters, age, and chronic health conditions. The results of this study provide supportive evidence for the observed association between the type of ventilator and mortality rates through multivariable analysis and reference to other studies.

## Conclusion

The study revealed that the utilization of non-ICU sophisticated ventilators was linked to a higher mortality rate when compared to standard ICU ventilators in COVID-19 patients with ARDS. However, given the shortage and limited availability of critical care equipment in ICUs during the COVID-19 pandemic, there is an inevitable need for transport ventilators in critical situations. To maximize the benefits of these ventilators, certain measures should be implemented, with the key factor being a trained professional operator. These measures encompass proper training, adherence to evidence-based guidelines, regular monitoring, and interdisciplinary collaboration. By adequately training professional operators, optimizing the utilization of ventilators, and implementing appropriate ventilation strategies, healthcare systems can provide optimal care during times of high demand. Nonetheless, it is important to emphasize that further multicenter studies are necessary to accurately determine the impact of different types of ventilators on the outcomes of critically ill patients.

## Methods

### Setting and ethical approval

This multi-center, retrospective observational study was conducted on 1,078 adult patients with ARDS due to COVID-19, who underwent MV at five university-affiliated hospitals in Iran (two hospitals in Tehran, Hamadan, Tabriz and Zahedan). The aim of the study was to evaluate the association between types of ventilators and their one-year survival rates. The study protocol was reviewed and approved by the Research Ethics Committees of Tabriz University of Medical Sciences, Tabriz, Iran, (IR.TBZMED.REC.1401.796), and study was conducted according to the principles of the Declaration of Helsinki^44^. Written informed consent from the patient or their relatives was required for participation in the original study. The manuscript was reported in according with the “Strengthening the Reporting of Observational studies in Epidemiology (STROBE) Statement”^45^.

### Study population

All Patients with ARDS due to COVID-19 admitted to five university-affiliated hospitals in Iran from March 2020 to April 2021 were enrolled in the study if they met all inclusion criteria. The inclusion criteria for participants in this study were as follows; (a) admitted patients of both genders over 18 years of age, (b) patients with moderate-to-severe ARDS, according to the Berlin definition^46^, which was defined as follows; patients with a BMI < 40 suffering from ARDS with PaO2/FiO2 < 300 mmHg during mechanically ventilated (MV) and an expected duration of controlled MV of more than 24 h, with the ability to tolerate Positive end-expiratory pressure (PEEP) titration, (c) ARDS due to proven COVID-19, confirmed by a positive result on a reverse-transcriptase–polymerase-chain-reaction (RT-PCR) assay of a specimen collected from a nasopharyngeal swab^47^, and (d) patients who received MV for more than 48 h. Patients excluded from the study if they (a) received MV for causes other than ARDS due to COVID-19, such as non-hypoxic cardiac arrest, cardiogenic or septic shock, neurological disorder, or pregnancy-related disease and (b) expressed unwillingness to participants in this study.

### Data collection

The data were extracted from the hospital registry including the demographic, clinical, chest computed tomography (CT) characteristics, mortality and ventilator type for each patient. Data on demographic information (age and gender), underlying diseases (yes or no), type of underlying diseases (pulmonary, cardiac or kidney failure), severity of illness based on Acute Physiology and Chronic Health Evaluation (APACHE) II^48^, which is calculated based on 12 physiological variables, including age, temperature, heart rate, respiratory rate, blood pressure, arterial pH, serum sodium, serum potassium, serum creatinine, hematocrit, white blood cell count, and Glasgow Coma Scale score. Each variable is assigned a score based on its deviation from normal values, and the scores are then added together to obtain a total score ranging from 0 to 71, duration of MV in hours, history of readmission (yes or no), and chest CT information following the Fleischner Society Nomenclature recommendations^49^. This includes details about the type of lesion (such as ground glass opacities [GGO], consolidation, crazy-paving pattern, GGO plus crazy paving, or GGO plus consolidation), other types of lesions (linear opacity, reversed halo sign, pleural effusion, intera lisional bronchiectasis, and lymphadenopathy), distribution of lesions (unilateral, bilateral, focal, and non-focal), presence of diffuse opacity, number of involved lobes, and an opacity scoring method implemented to stratify the degree of lung involvement. Each lobe was assigned a score between 0 and 5, with a maximum possible total score of 0 to 25. The scores were based on the percentage of involvement: score 0 (0% involvement), score 1 (less than 5% involvement), score 2 (5% to 25% involvement), score 3 (26% to 49% involvement), score 4 (50% to 75% involvement), and score 5 (greater than 75% involvement). The paragraph also mentions the type of ventilator used, which could be either an ICU ventilator or a transport ventilator.

### Protocols of treatment

- All patients received Remdesivir as an antiviral medication. They were given six doses over the course of 5 days.
- The patients also received dexamethasone, with a dosage ranging from 6–12 mg per day depending on the severity of their Acute Respiratory Distress Syndrome (ARDS).
- Mechanical ventilation was performed using volume Assist controlled ventilation with a tidal volume of approximately 6 ml/kg based on the patient's ideal body weight.
- If the plateau pressure exceeded 30 mmHg, the tidal volume was decreased by 1 ml/kg. This reduction could be repeated until reaching 4 ml/kg.
- The respiratory rate could be increased up to 35 breaths per minute depending on the levels of PaCO_2_ (partial pressure of carbon dioxide) and pH.
- Patients with a PaO2/FiO_2_ ratio less than 150 received cisatracurium at a rate of 30 mg/h for up to 48 h.
- In some cases, cisatracurium administration continued beyond 48 h due to factors such as dysynchrony (lack of coordination between patient and ventilator) and high respiratory rates, which were likely caused by the high level of sedation and analgesics administered.
- Sedation and analgesia were achieved using opioids and benzodiazepines with the goal of maintaining a Richmond Agitation-Sedation Scale (RASS) score of 0.0 to − 1, indicating a calm and lightly sedated state.
- None of the patients received Extracorporeal Membrane Oxygenation (ECMO), a technique that provides temporary heart and lung support in severe cases.

### Outcome

One-year survival, as the primary outcome, was assessed through telephone interviews conducted within the following 365 days for all patients. Patients were followed-up for a period of 1 year, and the final follow-up date was Jun 1, 2021. Additionally, in the event of a patient's death event occurred, that date of mortality was recorded.

### Statistical analysis

A descriptive analysis was conducted on all patients, as well as based on the type of ventilator used. For quantitative variables, mean ± standard derivation (SD) or median with interquartile range (IQR: 25–75%) were used, and comparisons were made using appropriate statistical tests such as Student’s t test or the Mann–Whitney U test. Categorical variables were expressed as percentage (%) and compared using the chi-square test or the Fisher’s exact test when applicable. Survival curves were generated using the Kaplan–Meyer method, and comparisons were made using the log-rank test and Cox model. Univariate and multivariate Cox proportional hazard regression analysis were performed to identify prognostic factors, particularly related to the type of ventilator and one-year survival rate. Hazard ratios (HRs) and 95% confidence intervals (CIs) were reported to compare hazards between patients' groups. To avoid over-fitting in the multivariate model, only factors with a p-value less than 0.2 in the univariate analysis were included. The final model was selected using forward conditional selection. All statistical analyses were performed using SPSS software (ver. 21) (SPSS Inc., Chicago, IL) and GraphPad Prism9© (GraphPad Software Inc., La Jolla, CA). A two-tailed *p*-value of < 0.05 was considered statistically significant in all analyses.

## Data Availability

All data collected and analyzed during the current study are available from the corresponding author on reasonable request.
